# What we think about professional and unprofessional behaviors: differences between the perception of clinical faculty members and medical students

**DOI:** 10.1186/s12909-022-03874-x

**Published:** 2022-12-14

**Authors:** Zahra Sadat Tabatabaei, Azim Mirzazadeh, Homayoun Amini, Mahboobeh Khabaz Mafinejad

**Affiliations:** 1 Department of Medical Education, School of Medicine, Tehran University of Medical Sciences, Tehran, Iran; 2 Education Development Office, School of Medicine, Mashhad University of Medical Sciences, Mashhad, Iran; 3 Department of Internal Medicine, School of Medicine, Tehran University of Medical Sciences, Tehran, Iran; 4 Department of Psychiatry, Roozbeh Hospital, Tehran University of Medical Sciences, Tehran, Iran; 5 Health Professions Education Research Center, Education Development Center, Department of Medical Education, Tehran University of Medical Sciences, Tehran, Iran

**Keywords:** Professionalism, Professional behaviors, Unprofessional behaviors, Perception, Clinical faculty members, Medical students

## Abstract

**Introduction:**

Differences in the viewpoints of clinical faculty members and medical students about prioritizing professional norms accepted by the professional community and lack of alignment of these views can lead to distortion of understanding, problems in learning and assessment of professionalism, and failure in students’ professional identity formation. This study aimed to identify the differences in viewpoints of clinical faculty members and medical students about prioritizing the importance and prevalence of professional and unprofessional behaviors among undergraduate medical students.

**Methods:**

A multi-stage qualitative study was conducted at Tehran University of Medical Sciences during 2020–2021. At first, a systematic search was conducted to identify professional and unprofessional behaviors using the directional content analysis method. A panel of experts was formed to check the codes obtained from reviewing the literature and to evaluate its compliance with the context. Then, the modified nominal group technique sessions were held with clinical faculty members and medical students to strengthen the codes extracted from the studies and systematically integrate their views to achieve a comprehensive list of professional and unprofessional behaviors in accordance with the context. Finally, a consensus was made among them about prioritizing the importance and prevalence of these behaviors in undergraduate medical students.

**Results:**

A total of 490 codes of professional behaviors and 595 unprofessional behavior codes were identified in the literature review. In the following sessions of the modified nominal group, 13 clinical faculty members listed 105 codes of professional and unprofessional behaviors, and 51 medical students also listed 313 codes. The results of the modified nominal group technique showed that the faculty members reported the importance of unprofessional behaviors higher than professional ones. At the same time, students rated the importance of professional behaviors higher than unprofessional ones. Both faculty members and students rate the prevalence of professional behaviors as high and the prevalence of unprofessional behaviors as low.

**Conclusion:**

The results showed a difference of views between clinical faculty members and medical students about prioritizing professional and unprofessional behaviors. It is essential to align their viewpoints to understand, learn and value professionalism to develop a professional identity.

## Introduction

Professionalism is considered one of the core competencies of medical students in medical curricula [1–3]. The Royal College of Physicians of England defines professionalism as a set of values, behaviors, and relationships that will strengthen patients’ trust in doctors [4]. Although professionalism may seem abstract, focusing on specific professional behaviors can help make it more tangible and practical in practice. These professional behaviors have been discussed in some articles as key elements of professionalism [5–7]. Undoubtedly, the existence of these professional behaviors in clinical faculty members and medical students and strengthening them is essential for improving the outcomes of medical care and maintaining patient safety [8].

In addition to identifying professional behaviors, focusing on recognizing and modifying of unprofessional behaviors is also vitally important for medical education [8–12]. Reconsidering and reflecting on unprofessional behaviors can lead to positive experiences [13]. Studies emphasize that clinical faculty members should also be aware of the importance of identifying and evaluating unprofessional behaviors [14, 15]. They also have a central role in encouraging and strengthening professional behaviors in medical students. If clinical faculty members fail to do so and do not respond appropriately to such behaviors, this message is implicitly conveyed to students that they do not care much about professionalism and that reforming unprofessional behaviors is unnecessary or not worth trying to change [7, 8, 13].

First and foremost, determining the critical elements involved in the concept of professionalism for these undergraduate medical students is essential to develop professional behaviors or modify unprofessional ones. However, reviewing the evidence shows that despite the commonalities in these key elements in different cultures, there are disagreements in the definitions presented in different communities. Therefore, it is imperative that each country and any institution develop its own definition of the professionalism of medical students in accordance with the social norms of the time of their community. They should also identify key elements of professionalism or express other professional or unprofessional behaviors of their undergraduate medical students formed due to the interaction between the individual and the context [4, 16–18]. To that end, Tehran University of Medical Sciences (TUMS) has developed a guideline on medical professional behaviors [19]. Teaching and assessing students’ professional behaviors in the educational program are based on these guidelines. Since The Professional Conduct Guide determines the norms of professions [4, 11], it is considered the basis for teaching and assessing subjects related to professionalism.

There are some factors such as population, ethnicity [11, 20], gender [21], various learning environments [12] and, generation differences [22] in the formation of a different understanding of professional and unprofessional behaviors. Most of these studies acknowledge the importance of developing a common language to describe professional behaviors in different generations by considering the context [22, 23]. This leads clinical faculty members and medical students to understand or share views about the professional norms adopted by their community [12]. Despite the importance of this issue, reviewing evidence continues to report a high prevalence of unprofessional behaviors in educational systems [11, 20, 24, 25]. Therefore, creating a shared view between clinical faculty members and medical students alone cannot be achieved by formulating a guideline for professional behaviors and determining unprofessional behaviors and evaluating them; it will require more detailed and coherent planning in this area. In this regard, it is necessary to consider systematic approaches to ensure the understanding and valuation of professionalism in medical education [4]. To the best of our knowledge, studies have not yet examined the differences between clinical faculty members’ and students’ views about prioritizing the importance of professional and unprofessional behaviors and their prevalence among undergraduate medical students using multi-stage qualitative methods. Therefore, our study aims to fill this gap.

## Method

Our qualitative study was conducted in a multi-stage design during 2020–2021. Usually, qualitative research is the best way to explore insight and perception [26]. In the first phase, a systematic search was conducted. Then, the consensus method was used to combine the viewpoints of clinical faculty members and students. One of the best consensus methods while reviewing studies for a structured scientific process is the Modified Nominal Group Technique (MNGT) [27]. In this study, this method was used to strengthen the extracted codes of studies and to systematically integrate the views of all participants to achieve a comprehensive list of professional and unprofessional codes of conduct in accordance with the context [10, 27]. Finally, with consensus between medical students and clinical faculty members, differences of views were determined regarding the importance of professional and unprofessional behaviors and their prevalence among undergraduate medical students.

### Stage 1: literature review

Initially, a systematic search was conducted to identify evidence related to medical students’ professional and unprofessional behaviors in pubMed, Eric, ProQuest and Scopus electronic databases. The articles were examined within a limited time frame from 2001 to 2020. The rationale for searching the literature within this time span was that almost all articles had been published between 2001 and 2020. From 2010 to 2020, research activity on professional and unprofessional behaviors increased in terms of volume, with 44 papers being published in 10 years, more than half of the total. Only articles in English were included in the study. Keywords included in the search strategy were (“Professional Behavior”, “Professional Behaviour”, “Professional norm”, “Professional ethic”, “Unprofessional Behavior”, “Unprofessional Behaviour”, “Unprofessional norm”, “Unprofessional ethic”, Medic*, Student, learner, Physician and Doctor).

The second phase of searching used ancestry searching (in the list of references to included studies) and forward tracing (in the citations of included studies) in Google scholar. Reference lists were also used manually. The search investigated various quantitative, qualitative, mixed-method, and review articles.

### Stage 2: expert panel

The list was then reviewed for two hours by the panel of experts considering two aspects: (1) Checking the accuracy of the codes categorized in the considered domains, and (2) Their conformity with the context and target groups of undergraduate medical students at TUMS (eliminated if not applicable). The expert panel, including 14 members, was drawn from faculty members in TUMS from different clinical specialties, including surgery, pediatrics, psychiatry, internal medicine, and obstetrics & gynecology. They were familiar with the issues of professionalism.

### Stage 3: modified nominal group technique

**Fig. 1:**
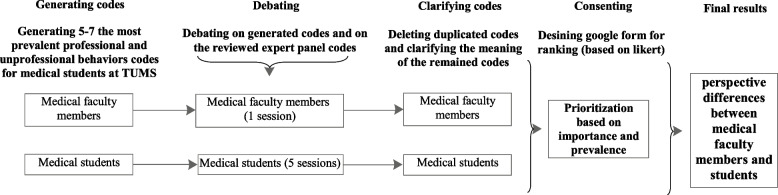
Modify Nominal Group Technique

The stages of the MNGT sessions were conducted for clinical faculty members and medical students according to Fig. 1 [27, 28].

Selecting faculty members to participate in the MNGT meeting was done based on purposive sampling, considering the criteria of their teaching in the undergraduate medical curriculum for at least five years and their familiarity with professionalism topics. The two-hour MNGT session was held in the presence of 13 faculty members from 9 specialized clinical disciplines. Also, five sessions of MNGT were held with medical students of clinical levels (clerkship and internship) with a total of 51 people. Purposive sampling was used to select medical students who have passed at least two semesters of their clinical phase and have passed the professionalism course. The participants’ specifications in the MNGT meetings are presented in Table 1.

**Table 1:** Demographic information of clinical faculty members and students are presented in the MNGT meetings

**Participants**	**characteristics**	**Number**
**Faculty members**	gender	Female: 8 Male: 5
	Discipline/fields	pediatrics:1 Psychiatry:1 Obstetrics & Gynecology: 2 Emergency medicine: 2 Cardiology: 1 Dermatology: 1 internal medicine: 3 Anesthesiology: 1 medical ethics: 1
	Teaching experrience	Between 5 to 10 years:10 Between 11 to 20 years:2 More than 20 years:1
**Medical students**	gender	Female: 28 Male: 23
	Average of age	Clerkship: 23 Internship: 25
	Clinical phase	Clerkship:17 Internship: 34
	Ethnicity	Native:27 Nonnative:24

The participants’ consensus was collected by an Internet form designed in Google Form based on a five-degree Likert scale in which score 1 means low and score 5 means high importance. The item mean and standard deviation for each ratings item were also calculated. Our selection criteria had the highest mean score in each domain, indicating a difference of view between clinical faculty members and medical students about prioritizing the importance and prevalence of professional and unprofessional behaviors.

## Results

### Literature review

**Fig. 2:**
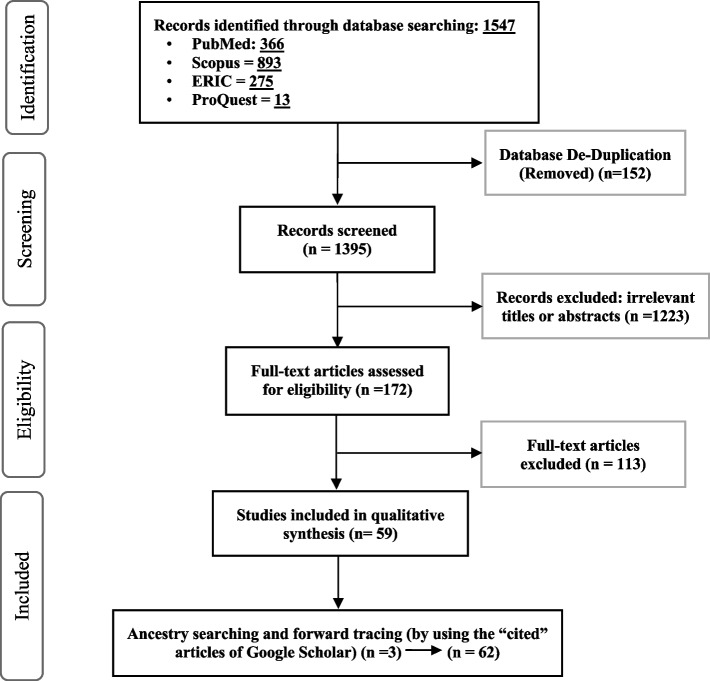
systematic search results (Jan. 2001 – Apr. 2020)

The details of the systematic search are shown in Fig. 2.

#### Extracted articles include

1. Quantitative, qualitative, or mixed-method articles that identify or evaluate the components of professional and unprofessional behaviors in medical students from the viewpoints of faculty members, patients, and other specialists.
2. Quantitative, qualitative, or mixed-method articles that identify or evaluate the components of professional and unprofessional behaviors of clinical faculty members, residents, or other medical students from medical students’ perspective.
3. Review articles and other studies that extensively consider students of different levels of medicine or medical sciences, physicians, and faculty members and identify or evaluate the components of professional and unprofessional behaviors.

Kappa coefficient (K score) was calculated as a measure of agreement. ZST and MKM are two authors that studied all topics and abstracts of the retrieved articles and extracted the related ones. The studies were reviewed by ZST and MKM and the agreement was 97% (1355/1395) with a kappa of 0.95 (CI = 0.847-1.0).

#### Frequency and content analyses

**Fig. 3:**
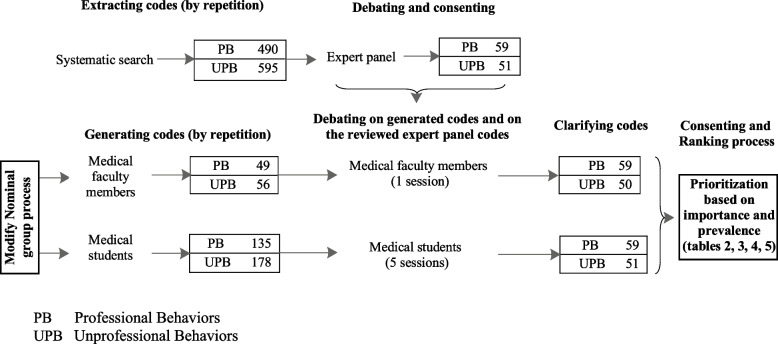
Results extraction Modified Nominal Group Technique

The analysis of the whole studies was performed by the directed content analysis method. The articles obtained during the analysis were read typicaly two times and in some cases three times. The codes were identified by considering the framework used in Saeedi Tehrani et al. in 2017 because the suggested framework was considered by the researchers to be comprehensive and compatible with the situation [19]. The codes were categorized into six domains: Honor and Integrity, Altruism, Excellence, Justice, Respect, and Responsibility. Code analysis was carried out by two researchers (ZST and MKM). Any difference or disagreement between them regarding the importance of prioritization and the prevalence of professional and unprofessional behaviors was discussed in two 2-hour sessions where the cases were reviewed and a consensus was reached. Then, the frequency analysis of each code of professional and unprofessional behaviors was determined, and a total of 490 codes (with repetition) of professional behaviors and 595 codes (with repetition) of unprofessional behaviors were identified (Table 2 and Fig. 3).

**Table 2:** Prioritizing the importance of professional and unprofessional behaviors for undergraduate medical students from the viewpoints of clinical faculty members and medical students and literature review

**Domains**	**Clinical faculty members (1 session)**		**Medical students (5 sessions)**		**Articles (62)**	
	**Professional behavior (Mean (SD))**	**Unprofessional behavior (Mean (SD))**	**Professional behavior (Mean (SD))**	**Unprofessional behavior (Mean (SD))**	**Professional behavior (Number)**	**Unprofessional behavior (Number)**
**Altruism**	4.04 (0.8)	4.02 (1.1)	4.28 (0.64)	4.1 (0.89)	84	26
**Honor and Integrity**	4.15 (0.78)	4.25 (0.8)	4.19 (0.69)	4.11 (0.94)	90	300
**Responsibility/ Conscientiousness**	4.14 (0.74)	4.4 (0.61)	4.14 (0.7)	4.13 (0.88)	81	77
**Respect**	4.22 (0.7)	4.49 (0.64)	4.16 (0.63)	4.11 (0.9)	103	91
**Justice**	4.08 (0.83)	4.27 (0.72)	4.2 (0.69)	4.1 (0.9)	24	19
**Excellence**	4 (0.9)	4.28 (0.8)	4.1 (0.65)	4.1 (0.94)	108	82

### Expert panel

In the expert panel, each code was reviewed and categorized in terms of conformity with the context, and the framework was modified during the expert panel. In this category, six codes were deleted, four binary codes were merged, four codes were corrected or clarified, and twenty-one were moved between domains (Fig. 3).

### Modified nominal group technique (MNGT)

#### Explaining participants’ perceptions and insights about professional and unprofessional behaviors in accordance with the context

At the beginning of the MNGT session, the participants were asked to produce 5–7 common professional and unprofessional behaviors in TUMS undergraduate medical students to evaluate their understanding and insights about professional and unprofessional behaviors based on the context and in response to an open question. On the whole, clinical faculty members produced 105 professional and unprofessional behavior codes (with repetitions), of which 49 codes (46.7%) were for professional behaviors, and 56 codes (53.3%) were for unprofessional behaviors among undergraduate medical students (Tables 3, 4 and 5). In the list developed by students, 313 codes of professional and unprofessional behaviors (with repetition) were produced in total, of which 135 codes (43.13%) were related to professional behaviors and 178 (56.87%) were those related to common unprofessional behaviors among undergraduate medical students (Tables 3, 4 and 5). The Generated codes by medical students and clinical faculty members were limited and referred to some of the output codes from the panel of experts (Tables 3, 4 and 5). Most of the generated codes by medical students and clinical faculty members in both professional and unprofessional behaviors were related to respect and the fewest number were related to justice (Table 3). Most of the codes extracted from studies were related to respect and excellence, and the fewest pertained to justice (Table 2). Then, after presenting the output codes from the panel of experts to the participants and after discussion, clarification and elimination of repetitive codes, only one code of unprofessional behaviors was removed by the clinical faculty members and four codes of professional behaviors were completed by the students.

**Table 3:** Prioritizing the prevalence of professional and unprofessional behaviors in undergraduate medical students from the viewpoints of clinical faculty members and medical students

**Domains**	**Clinical faculty members (1 session)**				**Medical students (5 sessions)**			
	**Professional behavior**		**Unprofessional behavior**		**Professional behavior**		**Unprofessional behavior**	
	**prevalence ranking (Mean (SD))**	**Generated code (Number)**	**prevalence ranking (Mean (SD))**	**Generated code (Number)**	**prevalence ranking (Mean (SD))**	**Generated code (Number)**	**prevalence ranking (Mean (SD))**	**Generated code (Number)**
**Altruism**	3.14 (0.95)	5	2.5 (1)	4	3.1 (0.9)	15	2.81 (1)	15
**Honor and Integrity**	3.16 (0.95)	8	2.2 (0.91)	15	3.18 (0.9)	22	2.3 (0.93)	48
**Responsibility/ Conscientiousness**	3.1 (0.9)	10	2.52 (0.76)	12	3.13 (0.91)	31	2.5 (0.95)	31
**Respect**	3.32 (0.92)	22	2.1 (1.1)	19	3.25 (0.92)	52	2.27 (0.98)	75
**Justice**	3.54 (1)	1	2 (0.9)	2	3.5 (0.79)	2	2.2 (0.93)	2
**Excellence**	2.92 (1.1)	3	2.65 (1.1)	3	2.8 (0.95)	13	2.68 (1)	5

**Table 4:** Production and prioritization of professional codes of conduct in terms of their importance and prevalence

**Domains**	**Code statement**	Clinical faculty members (1 session)			**Medical students (5 sessions)**		
		**Generated code (Number)**	**Importance ranking (Mean (SD))**	**prevalence ranking (Mean (SD))**	**Generated code (Number)**	**Importance ranking (Mean (SD))**	**prevalence ranking (Mean (SD))**
**Altruism**	Preferring patient's interests over one’s own personal interests	2	4.69 (0.63)	3.38 (0.77)	-	4.32 (0.74)	3.26 (0.72)
	Paying attention to the necessary safety measures for oneself and others	-	3.31(1.32)	3 (0.82)	-	4.54 (0.5)	2.78 (1.09)
	Cooperation with maximum power in case of urgent need of community to medical services in accidents	-	3.77 (0.93)	2.78 (1.17)	11	4.08 (0.7)	3.14 (0.89)
	Preserving the human dignity of the patient and his family	-	4.23 (0.6)	3.23 (1.01)	2	4.46 (0.5)	3.3 (0.84)
	Allocating enough time to listen patiently and actively to the patient and his/her family	3	4.69 (0.48)	3.54 (1.05)	-	4.22 (0.65)	2.95 (0.98)
	Assisting the healthcare team in solving professional problems and helping them in educational and research issues as much as possible	-	3.85 (0.69)	2.92 (0.86)	-	4.08 (0.72)	3 (0.88)
	Providing one’s counterparts and healthcare team with one’s knowledge and experiences	-	3.77(0.93)	3.15 (0.99)	2	4.3 (0.61)	2.98 (0.98)
**Honor and Integrity**	Observing honesty and trustworthiness in actions, speech, and writing	1	4.46 (0.66)	3.85 (0.9)	1	4.28 (0.57)	3.1(0.79)
	Accepting responsibility in case of medical errors and transferring one’s experiences in this field to the healthcare team	-	4.31(0.63)	2.69 (0.63)	-	4.22 (0.68)	2.56 (0.86)
	Getting help from faculty members, peers, and healthcare team in case of inability and lack of necessary skills to do one’s duties and informing the patient	1	4.38 (0.65)	3.15 (1.07)	1	4.24 (0.59)	3.1 (0.81)
	Not to offer information outside one’s scientific and practical skills and to refer people to experts	-	4 (0.58)	3.39 (0.96)	-	4.26 (0.6)	3.45 (0.94)
	Avoiding romantic relationship with the patient and his/her companions during treatment	-	4.33 (0.65)	3.58 (0.79)	3	4.28 (0.73)	4.02 (0.85)
	Observing professional boundaries and not abusing the patient or others for sexual, economic, advertising, or other such purposes	1	4.23 (0.83)	3.31 (1.03)	-	4.51 (0.54)	3.9 (0.9)
	Avoiding argument and physical confrontation with aggressive clients and informing the police	1	4.38 (0.65)	3.23 (0.93)	1	4.34 (0.59)	3.86 (0.76)
	Managing conflicts of interest in favor of patients' interests	-	3.92 (1.19)	3.31 (0.85)	-	4.14 (0.79)	3.1 (0.82)
	Keeping calm when one is tired, under pressure, and has personal problems	1	4 (0.91)	3.15 (0.8)	-	4.04 (0.76)	3.02 (1.01)
	Avoiding alcohol, drugs, and psychoactive substances in clinical and educational environments	-	4.46 (0.52)	3.15 (0.9)	-	4.24 (0.69)	2.88 (1.18)
	Observing the requirements of medical profession in one’s dressing code and behavior in clinical and academic environments	2	4.08 (1.04)	2.92 (1.26)	10	3.86 (0.84)	3.14 (0.87)
	Avoiding imposition of unnecessary costs on the patient and wasting health system resources and not abusing the authority and facilities of the system	1	4.08 (0.64)	2.92 (0.86)	-	4.17 (0.72)	2.96 (0.97)
	Not to abuse one’s authority	-	3.54 (1.2)	2.85 (0.9)	-	4.24 (0.72)	3.02 (1.07)
	Observing the rules, regulations, and ethical guidelines in all educational matters	-	4 (0.71)	2.85 (1.21)	6	4.1 (0.71)	2.88 (0.88)
	Observing general and specific ethical guidelines in all research affairs	-	4.08 (0.86)	3.08 (1.19)	-	4.08 (0.76)	2.9 (0.9)
**Responsibility/ Conscientiousness**	Using identification badge during the duty hours in such a way so as to be visible	1	4.23 (0.73)	3.77 (0.83)	2	3.85 (0.74)	3.23 (1.02)
	Availability and complete and timely fulfillment of responsibilities	3	4.38 (0.51)	3.46 (0.78)	18	4.29 (0.68)	3.29 (0.87)
	Transferring the responsibility of patient care to the qualified person after completing the shift	-	4.54 (0.52)	3.23 (0.83)	1	4.27 (0.64)	3.06 (1.02)
	Following up the treatment process and providing the patient with the necessary information to continue the treatment procedure after being discharged	-	4.42 (0.51)	2.77 (1.01)	4	4.31 (0.66)	3.21 (1.01)
	Timely and appropriate cooperation with the healthcare team and offering an integrated and continuous treatment plan	1	4.15 (0.69)	3.23 (0.73)	4	4.08 (0.65)	3.44 (0.85)
	Seeking help to provide services to patients in case of illness, personal problems, etc. and informing those in charge	-	3.85 (0.9)	2.62 (0.96)	-	4.25 (0.73)	3 (1.04)
	Providing feedback of professional misconduct or medical errors to colleagues and reporting them in serious cases and if they are not corrected	2	4.08 (0.76)	2.15 (0.9)	-	4.17 (0.66)	2.6 (0.96)
	Responding to professional expectations of faculty members, patients, etc. and accepting responsibility for one’s own professional and personal behaviors	2	4(1)	3.38 (0.87)	2	3.94 (0.74)	3.16 (0.65)
	Guiding the team effectively while training	1	3.62 (1.04)	3.31(1.18)	-	4.06 (0.77)	3.18 (0.8)
**Respect**	Providing clear information to the patient or his/her legal representative to obtain consent except in life threatening cases	-	4 (0.74)	3.08 (1.19)	5	4.33 (0.63)	3.27 (1.06)
	Respecting patient’s independence and the right to choose	-	4.23 (0.83)	3.08 (1.11)	4	4.1 (0.56)	2.98 (0.96)
	Treating the patient and his/her family with respect and sympathy while telling the truth	10	4.23 (0.6)	3.23 (0.99)	18	4.25 (0.57)	3.31 (0.93)
	Respecting the patient's right to choose another doctor and advising him or her in this regard	-	3.85 (0.99)	2.92 (0.87)	-	4.08 (0.68)	3.19 (1.09)
	Respecting patient's privacy	1	4.62 (0.51)	3.31 (0.78)	8	4.35 (0.67)	2.69 (1.26)
	Respecting patient's beliefs regardless of his/her ethnicity, religion, and culture	-	4.15 (0.69)	3.85 (1.13)	-	4.06 (0.56)	3.53 (0.83)
	Respecting patient’s dignity even when he/she is absent	-	4.15 (0.8)	3.62 (0.82)	-	4.25 (0.57)	3.04 (0.85)
	Observing confidentiality about patient information and not revealing his/her identity information	3	4.69 (0.48)	3.46 (0.77)	3	4.31 (0.72)	3.25 (0.84)
	Preserving human dignity in the dissection room and other learning situations	-	4.46 (0.66)	3.54 (0.85)	-	4.17 (0.75)	3.33 (0.81)
	Not criticizing the decisions of the healthcare team in the presence of patients and their companions and introducing the legal follow-up procedures if requested by the patient	-	4.31 (0.63)	3 (1.19)	-	4.27 (0.65)	3.25 (0.92)
	Respecting the role and skills of the members of the healthcare team and honest and patient interaction while observing human dignity and hierarchy	7	4.08 (0.64)	3.38 (1.11)	9	3.98 (0.6)	3.5 (0.62)
	Respect and gratitude to faculty members and more experienced students	-	3.92 (0.86)	3.42 (0.99)	5	3.73 (0.64)	3.6 (0.85)
**Justice**	Observing religious laws and regulations of the medical profession while considering academic rules	-	3.92 (0.76)	3.42 (1.11)	-	4 (0.65)	3.46 (0.77)
	Avoiding any discrimination based on age, gender, literacy, nationality, ethnicity, race, language, beliefs, criminal background, and socioeconomic status	-	4.15 (0.8)	3.69 (1.11)	2	4.38 (0.6)	3.72 (0.76)
	Paying attention to the quality and comprehensiveness of services to vulnerable groups like other patients and respecting their rights and dignity	1	4.23 (0.8)	3.69 (1.03)	-	4.38 (0.57)	3.8 (0.76)
	Serving people with special diseases in accordance with safety standards and principles like other patients	-	4.15 (0.8)	3.69 (0.95)	-	4.28 (0.64)	3.52 (0.76)
	Treating the healthcare team fairly and trying to resolve differences between them while considering patient dignity	-	4 (0.82)	3.58 (0.88)	-	4.1 (0.81)	3.26 (0.88)
	Using fair criteria and tools in evaluating faculty members and colleagues and avoiding bias	-	4 (1)	3.15 (0.99)	-	4.02 (0.85)	3.06 (0.88)
**Excellence**	Awareness of one’s strengths and weaknesses and reflecting on their own actions and behaviors	-	4.33 (0.65)	2.92 (1.09)	-	4.31 (0.62)	3.06 (0.95)
	Listening to the opinions and feedbacks of the healthcare team and patients about their professional behaviors and abilities and reflecting on them	2	4.08 (0.86)	2.85 (0.99)	1	3.88 (0.61)	2.65 (0.98)
	High initiative and motivation for excellence and promotion	1	4.38 (0.77)	2.92 (1.19)	9	4 (0.72)	2.77 (0.99)
	High intellectual maturity in dealing with crises and using self-management mechanisms and strong decision-making	-	4.08 (0.76)	2.92 (0.87)	-	4.44 (0.54)	3 (0.92)
	Being interested in the medical profession, having a strong and confident personality, and accepting assigned responsibilities in different situations	-	4 (0.91)	2.54 (0.88)	1	4.17 (0.66)	2.96 (0.99)
	Accepting logical reasoning in discussions with faculty members, peers, etc. and avoiding belligerent, destructive and unfounded criticism	-	4.23 (0.83)	3.15 (1.14)	2	4.33 (0.6)	3.45 (0.88)
	Timely and effective guidance and feedback to peers and healthcare team and providing constructive suggestions for better learning and performance improvement	-	3.92 (0.76)	3.23 (1.17)	-	4.02 (0.53)	2.77 (0.99)
	Promoting physical, mental, and social health by adjusting lifestyle in issues such as daily habits, recreation, nutrition, disease prevention, etc	-	3.92 (1.04)	2.77 (1.17)	-	4.29 (0.68)	2.1 (0.93)
	Cooperation with the formal evaluation process conducted by the university or other competent authorities of one’s health status, behavior and performance	-	3.62 (1.19)	3.23 (1.17)	-	3.73 (0.71)	3.17 (0.95)
	Referring to the hospital's clinical administration office in case of a complaint	-	3.46 (1.33)	2.69 (1.18)	-	3.88 (0.9)	2.27 (0.84)

**Table 5:** Production and prioritization of unprofessional codes of conduct in terms of their importance and prevalence

**Domains**	**Code statement**	**Clinical faculty members (1 session)**			**Medical students (5 sessions)**		
		**Generated code (Number)**	**Importance ranking (Mean (SD))**	**prevalence ranking (Mean (SD))**	**Generated code (Number)**	**Importance ranking (Mean (SD))**	**prevalence ranking (Mean (SD))**
**Altruism**	Preferring one’s personal interests over those of the patients	2	4.33 (0.98)	2.54 (0.78)	4	3.98 (1.02)	2.65 (0.91)
	Ignoring necessary safety measures for oneself and others	-	3.75 (1.22)	2.31(0.85)	-	4.29 (0.74)	3.48 (1.13)
	Not listening patiently and actively to the patient or his/her family and not allocating enough time to them	2	4.17 (0.83)	2.77 (1.09)	10	4.22 (0.87)	2.65 (1.06)
	Not sharing one’s knowledge and experiences with one’s peers and healthcare team	-	3.83 (1.27)	2.38 (1.39)	1	3.96 (0.92)	2.4 (1.01)
**Honor and Integrity**	Lack of honesty and trustworthiness in actions, speech, and writing	3	4.33 (0.89)	2.46 (1.05)	11	4.17 (0.91)	2.52 (0.95)
	Providing information outside one’s scientific and practice skills and not directing patients to experts	-	4.25 (0.87)	2.31 (1.25)	3	4.19 (0.89)	2.17 (0.95)
	Not accepting responsibility in case of medical errors and not transferring one’s experiences to the healthcare team	-	4.08 (0.51)	2.62 (0.87)	4	4.1 (1.04)	2.77 (0.93)
	Not asking for help from faculty members, peers, and healthcare team when one is unable to take care of the patient or lacks the required skills and not informing the patient about such matters	-	4.17 (0.94)	2.67 (1.13)	6	4.19 (0.92)	2.06 (0.89)
	Establishing a romantic relationship with the patient and his/her companions during treatment	-	4.09 (0.83)	1.58 (0.67)	1	4.15 (0.92)	1.38 (0.61)
	Not observing professional boundaries and abusing the patient or others for sexual, economic, advertising, or other such purposes	-	4.17 (0.83)	1.69 (0.85)	1	4.25 (0.96)	1.71 (0.92)
	Creating discord between peers and other members of the healthcare team and provoking challenging, authoritarian and negative behaviors	2	3.92 (1.24)	2.08 (0.95)	2	4.08 (0.87)	2.19 (0.94)
	Verbal argument and physical confrontation in dealing with the client's violence and not informing the police	-	4.17 (0.83)	1.85 (0.99)	2	3.9 (0.91)	1.92 (0.68)
	Failure to manage conflict of interests in favor of patients' benefits	-	4.08 (1.08)	2.23 (0.73)	3	4.13 (0.96)	2.17 (0.91)
	Not keeping calm when tired, under job stress or when having personal problems	-	4.33 (0.65)	2.77 (0.93)	1	4.21 (0.92)	2.93 (1.07)
	Using alcohol, drugs, and psychoactive substances in clinical and educational environments	1	4.75 (0.45)	1.92 (0.86)	1	4.17 (1.04)	2.73 (1.38)
	Not observing the norms of the medical profession in one’s dressing habit and behavior in clinical and academic environments	6	4.36 (0.67)	2.58 (1.165)	9	3.94 (0.86)	2.35 (0.93)
	Unhealthy and counterproductive competition with peers in educational/research/clinical situations	-	4.25 (0.75)	2.31(1.03)	-	3.96 (0.92)	2.35 (0.76)
	Imposing undue costs to patients and wasting health system resources and not abusing the authorities and facilities of the health system	1	4.25 (0.87)	2.23 (0.83)	2	4.06 (1)	2.38 (1.3)
	Abusing one’s authority	2	4.25 (0.87)	1.77 (0.73)	1	4.17 (0.98)	2.23 (1.04)
	Ignoring the rules, regulations, and ethics guidelines in educational matters	-	4.42 (0.67)	1.92 (0.76)	1	4.17 (0.86)	2.35 (0.86)
	Ignoring general and specific ethical guidelines in all research	-	4.42 (0.67)	2.46 (0.66)	-	4.08 (0.85)	2.5 (0.97)
**Responsibility/ Conscientiousness**	Failure to fulfill full responsibilities on time and not being available	9	4.75 (0.45)	2.62 (0.77)	24	4.11 (1)	2.61 (0.98)
	Not following the treatment process and not providing the necessary information after discharge	1	4.5 (0.52)	2.92 (0.64)	4	4.16 (0.9)	2.63 (1.04)
	Lack of timely and appropriate cooperation with the healthcare team and not providing an integrated and continuous treatment plan	2	4.17 (0.83)	2.42 (0.67)	2	4.15 (0.8)	2.29 (0.87)
	Not asking for help in providing services to patients in case of illness or personal problems, etc. and not informing the person in charge	-	4.25 (0.62)	1.92 (0.86)	-	4.21 (0.77)	2.75 (1.02)
	Not providing the colleagues with feedback of professional misconduct or medical errors and not reporting serious cases and failing to correct the behavior	-	4.17 (0.58)	2.23 (0.83)	-	4.06 (0.84)	2.38 (0.87)
	Not being accountable to professional expectations of faculty members, patients, etc. and not taking responsibility for one’s professional and personal performance	-	4.58 (0.67)	3 (0.82)	1	4.15 (0.81)	2.32 (0.86)
**Respect**	Failure to provide clear information to the patient or his/her legal representative for obtaining consent	-	4.58 (0.52)	1.85 (0.9)	5	4.14 (0.94)	2.43 (1.24)
	Lack of respect for the patient’s independence and right to choose	-	4.17 (0.94)	2.31 (0.95)	3	4.12 (1.01)	2.51 (1.02)
	Lack of respect and sympathy with the patient and his family when telling the truth	6	4.33 (0.78)	1.69 (0.75)	19	4.1 (0.93)	2.38 (0.87)
	Not respecting the patient's privacy	4	4.67 (0.49)	2.46 (1.45)	12	4.21 (0.97)	2.96 (1.27)
	Lack of respect for patients' beliefs of different ethnicities, religions, and cultures	-	4.67 (0.65)	1.69 (0.95)	-	4.06 (0.81)	1.85 (0.8)
	Lack of respect for the patient's dignity, even in his/her absence and his/her dignity in the dissecting room	-	4.58 (0.51)	2.08 (0.86)	3	4.1 (0.91)	2.42 (0.85)
	Not observing confidentiality about patient’s information and his/her identity information	6	4.67 (0.49)	2.23 (1.42)	19	4.23 (0.88)	2.31 (0.97)
	Not observing confidentiality about the information of faculty members /peers/ healthcare team and revealing their identity information	3	4.75 (0.45)	2.31 (1.38)	3	4.08 (0.9)	2.31 (0.78)
	Criticizing the decisions of the healthcare team in the presence of patients/their companions and disrespecting them	-	4.5 (0.52)	2.08 (1.04)	4	4.23 (0.78)	2.13 (1.12)
	Lack of respect for the role and skills of the members of the healthcare team and lack of honest and patient interaction based on human dignity and ignoring the hierarchy	-	4.33 (0.65)	1.92 (0.86)	5	4.02 (0.79)	2.04 (1.05)
	Not showing respect and gratitude towards the more experienced faculty members and students	-	4.18 (0.98)	2.31 (1.18)	2	3.88 (0.89)	1.83 (0.86)
**Justice**	Ignoring religious laws and regulations of the medical profession and academic rules	-	4.09 (0.7)	1.92 (0.86)	-	3.9 (0.92)	2.27 (0.86)
	Discrimination based on age, gender, literacy, nationality, ethnicity, race, language, beliefs, criminal background, and socioeconomic status	1	4.42 (0.67)	1.77 (1.01)	2	4.19 (0.96)	1.92 (0.85)
	Failing to serve people with special diseases in accordance with safety standards and principles used for other patients	-	4.67 (0.49)	2.15 (0.9)	-	4.21 (0.97)	2.02 (0.91)
	Unfair behavior toward the healthcare team and not trying to resolve disputes between them while observing the human dignity	1	4.27 (0.65)	2.08 (0.95)	-	4.08 (0.8)	2.56 (0.94)
	Not using fair criteria and tools in evaluating faculty members and colleagues and showing partiality	-	3.92 (1.08)	2.08 (0.79)	-	3.96 (0.8)	2.51 (1.06)
**Excellence**	Ignorance of one’s strengths and weaknesses and lack of reflection on one’s actions and behaviors	1	4.5 (0.8)	2.62 (1.19)	2	4.13 (0.96)	2.77 (0.93)
	Not paying attention to the opinions and feedbacks of the healthcare team and patients about one’s professional behaviors and abilities and not reflecting on them	1	4.5 (0.52)	2.69 (1.25)	-	4.08 (0.92)	2.48 (0.88)
	Poor initiative and inner motivation for one’s excellence and all-round promotion	1	4.08 (0.67)	3 (1)	1	4.04 (0.9)	2.71 (0.97)
	Poor intellectual maturity in dealing with crises and not using self-management and strong decision-making mechanisms	-	4.33 (0.78)	2.69 (0.95)	2	4.06 (0.89)	2.63 (1.04)
	Lack of interest in medical profession, deficient personality, poor self-confidence and not accepting one’s responsibilities in different situations	-	4.17 (1.03)	2.77 (1.09)	-	4.1 (0.95)	3.02 (1.1)
	Rejecting logical reasoning in discussions with faculty members, peers, etc. and showing hostile, malicious and unfounded criticism	-	4.55 (0.52)	2.23 (0.83)	-	4.13 (0.89)	2.33 (0.91)
	Not providing guidance and timely and effective feedback to peers and healthcare team and not making constructive suggestions for better learning and performance improvement	-	3.82 (1.33)	2.58 (1.08)	-	3.92 (0.94)	2.75 (1)

#### Prioritizing views between the two groups of participants

Finally, with the consensus of participants, the differences of viewpoints were identified between clinical faculty members and medical students about prioritizing the importance and prevalence of professional and unprofessional behaviors among undergraduate medical students of TUMS. Also, the difference between frequency analysis of articles and behaviors developed by the clinical faculty members and the medical students regarding professional and unprofessional behaviors among undergraduate medical students was determined. The results can be seen in Tables 2, 3, 4 and 5.

##### Importance

From the professional behavior codes extracted from the studies, the code with the most repetition belonged to the domain of Excellence and then the domain of Respect. Regarding unprofessional behavior, the codes with the most repetition belonged to the domain of Honor and Integrity and then the domain of Respect (Please see Table 2). It is also noteworthy that the number of professional and unprofessional behavior codes generated by both groups of faculty members and students at the beginning of MNGT belonged to the domain of Respect (Please see Table 3). Moreover, in prioritizing the importance of professional and unprofessional behavior, from the point of view of clinical faculty members, the domain of Respect was given the highest priority (Please see Table 2). Furthermore, in prioritizing the importance of professional behavior, from the students’ point of view, the domain of ​​Altruism and unprofessional behavior and the domain of ​​Responsibility/conscientiousness were given the highest priority (Please see Table 2). The most essential codes prioritized by clinical faculty members are related to unprofessional behaviors, and the less important prioritized codes are related to professional behaviors. Consequently, clinical faculty members understand the importance of unprofessional behaviors more than professional ones (Tables 4 and 5). Also, the most important codes prioritized by medical students are related to professional behaviors and the prioritized codes with low importance are related to unprofessional behaviors. Therefore, unlike the clinical faculty members, students consider the importance of professional behaviors more than unprofessional ones (Tables 2 and 3). Studies show that out of 1085 of the identified and extracted codes (total number of professional and unprofessional behaviors codes with repetitions), 595 (54.84%) codes of behaviors are related to unprofessional ones (Table 2).

##### Prevalence

Regarding the prioritizing of the prevalence of professional behaviors by both clinical faculty members and medical students, the domain of Justice was given the highest priority. In prioritizing the prevalence of unprofessional behaviors, from the point of view of clinical faculty members, the domain of Excellence had the highest prevalence, and according to medical students, the domain of Altruism had the highest prevalence (Please see Table 3). Furthermore, in prioritizing the prevalence of unprofessional behaviors in each group of clinical faculty members and medical students, the domain of Justice had the lowest prevalence (Please see Table 3). The codes prioritized with high prevalence by clinical faculty members and medical students are related to professional behaviors and those prioritized by them with low prevalence are related to unprofessional behaviors. As a result, both clinical faculty members and medical students rate the prevalence of professional behaviors in undergraduate medical students as high and the prevalence of unprofessional behaviors as low. However, differences of views about prioritizing the prevalence of professional and unprofessional behaviors are visible in most codes (Tables 3 and 5).

## Discussion

Identifying professional and unprofessional behaviors in clinical faculty members and medical students and strengthening them is essential for improving the outcomes of medical care [8]. Our study proposes a process to assess the differences between clinical faculty members’ and students’ views on prioritizing the importance and prevalence of professional and unprofessional behaviors by creating consensus. The aim is to bring these views closer to each other to promote and strengthen professional behaviors.

Previous studies have primarily focused on identifying and describing professional and unprofessional behaviors [8, 18] or evaluating physicians, medical students, and clinical faculty members’ perceptions of professional and unprofessional behaviors in the form of quantitative studies and qualitative interviews [2, 12, 15, 19, 29]. Some of these studies examine factors affecting professional and unprofessional behaviors such as population, ethnicity [11, 20], gender [21], different learning environments [12] and generational differences in professional perception [22]. There are also articles on evaluation strategies and methods for diagnosing and correcting unprofessional behaviors in students [9, 13, 30, 31]. Other examples of these strategies include detecting abusive behaviors [32] or addressing student characteristics associated with an increased risk of professional misconduct [33, 34]. Several other studies have also been conducted to help faculty members identify unprofessional behaviors in undergraduate medical students. By presenting a model, these studies facilitated the identification of students who performed poorly in professional skills [10, 35]. However, according to our studies, no existing research has investigated the differences of views about prioritizing the importance and prevalence of professional and unprofessional behaviors and identifying different priorities of such behaviors between clinical faculty members and medical students by creating consensus. In this study, the overall view of the results obtained in the prioritization of the importance of professional and unprofessional behaviors indicates that clinical faculty members have reported the importance of unprofessional behaviors above professional ones. On the contrary, students have reported the importance of professional behaviors higher than unprofessional ones. In prioritizing the prevalence of professional and unprofessional behaviors, faculty members and medical students reported the prevalence of professional behaviors higher than the prevalence of unprofessional ones; however, in prioritizing the prevalence of codes, some differences of view were visible among them. Reporting a higher prevalence of professional behaviors than unprofessional ones by clinical faculty members can have several reasons. For instance, although clinical faculty members observe unprofessional behaviors in 20% of all medical students, they only report 3–5% [8, 36–38]. Another reason is the greater tendency of clinical faculty members to report positive findings because focusing on positive behaviors may be more effective in learning [39, 40]. Finally, other possible reasons include a lack of transparent criteria for unprofessional behaviors (lack of professionalism) in medical school, concerns about the subjectiveness of one’s judgment, and concerns about damage to student credibility [8, 41]. However, it seems that focusing on unprofessional and professional behaviors is part of learning. Clinical faculty members should be prepared to identify these behaviors more accurately and provide feedback to the students [8]. Their lack of response to medical students’ unprofessional behaviors means that being unprofessional is acceptable and that responding to it is unnecessary [7, 8, 13].

### Altruism domain

A closer look at prioritizing codes of conduct by clinical faculty members and medical students shows that students have assigned the highest importance to the altruism domain in prioritizing professional codes of conduct and the highest prevalence in prioritizing unprofessional behaviors codes. One of the most important codes indicating the difference of views between clinical faculty members and medical students was “paying attention to the necessary safety measures for oneself and others”. This code was the most important code of professional and unprofessional behaviors prioritized by medical students and the least important code of such behaviors prioritized by clinical faculty members from all domains. The results can be interpreted in this way: clinical faculty members are usually less concerned about this issue due to multiple and frequent encounters with clinical situations associated with lack of equipment and facilities and because of formation of their professional identity in providing services to patients. However, students show more concern in this respect due to the training received during their studies and little experience. Also, medical students assigned the highest rank to this code in prioritizing the prevalence of unprofessional behaviors. This finding did not align with the results of the 2020 McGurgan et al. study in which medical students were asked about the acceptability of a range of professional behaviors in challenging situations, whether they faced similar situations, and what measures they would take in those situations. Of 1413 students who were involved with real-life infectious diseases, 98.5% agreed with not attending clinical settings in these conditions. Also, out of 1473 people who encountered lack of personal equipment, 97.9% stated that they agreed with refraining from continuing to work under these conditions [11], indicating a low prevalence of this unprofessional behavior among students.

### Honor and integrity domain

Most of the codes identified in the literature review phase of our study discuss unprofessional behaviors related to the domain of honor and integrity. In similar cases, Ainsworth’s article in 2018 showed that the most reported unprofessional behaviors pertained to the domain of integrity [42]. Also, the code “observing honesty and trustworthiness in actions, speech and writing” related to the domain of honor and integrity was one of the highest prioritized codes with high prevalence according to clinical faculty members. Despite clinical faculty members’ lower priority reports of unprofessional behaviors among undergraduate medical students in this domain in general, in some codes of professional and unprofessional behaviors here, clinical faculty members reported a higher prevalence of unprofessional behaviors. For example, one can refer to the following code: “Not asking for help from clinical faculty members, peers and healthcare team when one is unable to take care of the patient or lacks the required skills and informing the patient about such matters.“ Students considered unprofessional behaviors such as “alcohol, drugs and psychoactive consumption in clinical settings” to be more prevalent as compared with clinical faculty members’ views on the same topic. Perhaps the reason for that is the student’s knowledge of and relationship with each other, both in and outside the clinical setting. In a similar study conducted by Brockbank et al. in 2011, people, medical students, and physicians rated the unprofessional behavior “criminal activity and drug abuse” more seriously than other unprofessional behaviors [43]. In 2017, Cullen et al. expressed the greatest concern about unprofessional behaviors in the integrity domain. The study stated some of the related unprofessional behaviors, like “showing clear signs of substance abuse,“ are critical even if they happen once [32].

### Respect domain

As for respect, the extracted codes of the literature review phase and the codes produced by clinical faculty members and medical students in the present study were higher than other domains. Several studies show that students were more willing to express experiences related to respect than other types of experiences [3, 13, 44] or ranked them among the highest. In this regard, the study by Byszewski et al. in 2012 showed that in the ranking of professional behaviors by students, the highest rank was related to the respect domain [45]. Also, most of the produced codes related to unprofessional behaviors by medical students were in the domain of respect. In this regard, Cuesta-Briand et al. also stated in their 2014 study that students are more inclined to describe examples of “unacceptable” or “unprofessional” behaviors observed in clinical settings [46] when they discuss the importance of respectful treatment of patients and colleagues. However, it is worth noting in our study that there was a distinct difference between clinical faculty members and medical students in prioritizing the importance and prevalence of behaviors in this domain, especially in the field of unprofessional behaviors.

One of the cases where clinical faculty members reported a higher prevalence of unprofessional behaviors than medical students in their prioritization is a “lack of respect for clinical faculty members and more experienced students.“ These can indicate higher expectations of clinical faculty members in observing professional behaviors in interaction with seniors and clinical faculty members. In addition, students’ attention and respect for the position of clinical faculty members can be one of the reasons emphasized in the culture of Eastern societies because of the valuable position of clinical faculty members in the process of further education by teachers and educators. Besides, the difference of perspective in defining instances of respect can be effective because of the generational difference between clinical faculty members and medical students.

### Responsibility/conscientiousness domain

In the domain of responsibility/conscientiousness, the codes extracted from the literature review phase of the present study and the codes produced by clinical faculty members and medical students were more moderate as compared to other domains. In contrast to our research, Cullen et al. in 2017 considered the field of conscientiousness as creating the least concern in unprofessional behaviors [32]. In ranking the importance and prevalence of professional and unprofessional behaviors, the proximity of medical students’ and clinical faculty members’ views in this domain was more than other areas. In this regard, Mak-Van Der Vossen et al. believe that the views of faculty members and medical students regarding their duties and responsibilities can be improved by strengthening the teamwork among them [18]. Regarding the code “Not asking for help in providing services to patients in case of illness or personal problems, etc. and not informing the person in charge, “we can point to the significant difference of opinion in the discussion of prioritizing the importance of professional behaviors. Clinical faculty members have considered less important than medical students while students have prioritized the prevalence of this unprofessional behavior over clinical faculty members. One of the reasons for the report of the prevalence of unprofessional behaviors in the domain of responsibility and conscientiousness from the point of view of medical students can be this: Because medical students at TUMS have perfectionist tendencies in accepting responsibilities and performing duties, they may have higher professional expectations in the domain of conscientiousness and professional responsibilities.

### Justice domain

In the domain of justice, the codes extracted from the literature review phase of the present study and the codes produced by clinical faculty members and medical students were very few. This finding is similar to the results of a 2014 study by Al-Abdulrazzaq et al. In that study, out of the total professional behaviors listed by medical students, justice was one of the lowest domains listed [44]. One reason for this could be that the word justice has conceptual complexities and it is difficult for people to determine the codes associated with this domain. In prioritizing the importance and especially the prevalence of professional behaviors, the viewpoints of clinical faculty members and medical students from all other domains are very close together. According to medical students and clinical faculty members, the prevalence of justice, compared with other domains, is of high priority in professional behaviors and lower priority in unprofessional ones.

### Excellence domain

In our study, in the domain of excellence, codes extracted from the literature review phase were numerous; however, the codes produced by clinical faculty members and medical students were very few. From the viewpoint of medical students and clinical faculty members, the importance of excellence in prioritizing professional and unprofessional behaviors was low. Its prevalence in prioritizing professional and unprofessional behaviors was low and high, respectively. In 2017, Cullen et al. expressed less concern from the perspective of experts about unprofessional behaviors in this domain [32]. The low prevalence of codes in this domain in the discussion of professional behaviors and its high prevalence in unprofessional ones, as reported by medical students and clinical faculty members, can be due to the fact that the issues related to excellence have not been systematically addressed in the curriculum. In addition, the low prevalence of these professional behaviors in medical students can be the result of inadequate recognition of the instances related to professional excellence among clinical faculty members and medical students due to the newness of the subject and only addressing the issue in the medical education literature in recent years [18, 47].

Ideally, professional behaviors are developed in collaboration with clinical faculty members and medical students [8, 48]. In this regard, clinical faculty members should create a clear (implicit and explicit) concept for themselves and their students by carefully planning and empowering themselves regarding existing professional and unprofessional behaviors based on the context. By determining transparent, professional expectations, professional and unprofessional behaviors can be evaluated in medical students and clinical faculty members using standards that will ultimately lead to a fair assessment and effective formative learning experience [8, 18]. Also, by clarifying professional and unprofessional behaviors and discussing them with clinical faculty members and medical students, in addition to understanding these behaviors better, conflicts between them will be resolved and a common dialogue will be formed between them [18, 49]. Therefore, in addition to adequately understanding professional and unprofessional behaviors in accordance with the field, medical students will learn how to strengthen professional behaviors and avoid being unprofessional.

Some limitations of this study that should be considered are the small sample size. A limitation of the method we used is that the participants who attended in one MNGT for clinical faculty members and five MNGT for medical students did not interact with each other. Thus, they were not able to comment on ideas from other groups. We addressed this limitation by performing a member checking of the combined results of all MNGTs. Further research is needed to identify differences of views about prioritizing the importance and prevalence of professional and unprofessional behaviors among managers and graduates. Also, it is essential to effectively plan for aligning the views for learning and valuing the principles of professionalism and the correct formation of professional identity in undergraduate medical students.

## Conclusion

Our study showed that clinical faculty members reported the importance of unprofessional behaviors as higher than professional ones, while medical students, on the contrary, considered the importance of professional behaviors as higher than unprofessional ones. In prioritizing the prevalence of professional and unprofessional behaviors in undergraduate medical students, clinical faculty members and medical students reported the prevalence of professional behaviors higher than the prevalence of unprofessional ones. However, differences of views about prioritizing the prevalence of professional and unprofessional behaviors were visible in most codes. Also, the most important code of professional and unprofessional behaviors, as prioritized by medical students, turned out to be the least important code ranked by clinical faculty members. Therefore, identifying these differences of views about ranking the importance and prevalence of professional and unprofessional behaviors and aligning them among clinical faculty members and medical students leads to understanding, learning and valuation of professionalism principles and ultimately leads to the correct formation of professional identity in undergraduate medical students.

## Data Availability

The datasets used and/or analyzed during the current study are available from the corresponding author upon request.
